# Histone modification‐linked prognostic model for ovarian cancer reveals LBX2 as a novel growth promoter

**DOI:** 10.1111/jcmm.18260

**Published:** 2024-03-23

**Authors:** Jian Xiong, Hongyuan Liang, Xiang Sun, Kefei Gao

**Affiliations:** ^1^ Department of Obstetrics and Gynecology, Guangzhou Women and Children's Medical Center Guangzhou Medical University Guangzhou China; ^2^ Department of Radiology Shengjing Hospital of China Medical University Shenyang China

**Keywords:** immune infiltration, LBX2, ovarian cancer, prognosis, proliferation

## Abstract

Ovarian cancer (OC) is a deadly disease with limited treatment options and poor overall survival rates. This study aimed to investigate the role of histone modification‐related genes in predicting the prognosis of OC patients. Transcriptome data from multiple cohorts, including bulk RNA‐Seq data and single‐cell scRNA‐Seq data, were collected. Gene set enrichment analysis was used to identify enriched gene sets in the histone modification pathway. Differentially expressed genes (DEGs) between histone modification‐high and histone modification‐low groups were identified using Lasso regression. A prognostic model was constructed using five selected prognostic genes from the DEGs in the TCGA‐OV cohort. The study found enrichment of gene sets in the histone modification pathway and identified five prognostic genes associated with OC prognosis. The constructed risk score model based on histone modification‐related genes was correlated with immune infiltration of T cells and M1 macrophages. Mutations are more prevalent in the high‐risk group compared to the low‐risk group. Several drugs were screened against the model genes. Through in vitro experiments, we confirmed the expression patterns of the model genes. LBX2 facilitates the proliferation of OC. Histone modification‐related genes have the potential to serve as biomarkers for predicting OC prognosis. Targeting these genes may lead to the development of more effective therapies for OC. Additionally, LBX2 represents a novel cell proliferation promoter in OC carcinogenesis.

## 1 INTRODUCTION

Ovarian cancer (OC) is a significant health concern, with a high mortality rate and limited treatment options.^1^ Currently, various strategies have been developed for the prevention and treatment of OC. For individuals at high‐risk, such as those with a family history of OC or carriers of BRCA1/BRCA2 gene mutations, prophylactic oophorectomy is recommended. Early‐stage patients may undergo comprehensive staging surgery, while late‐stage patients may undergo cytoreductive surgery. Following surgery, patients typically receive combination chemotherapy based on platinum agents, such as cisplatin/carboplatin plus paclitaxel, cisplatin/carboplatin plus doxorubicin, or other specific chemotherapeutic agents, to eradicate tumour cells. For recurrent or refractory OC, radiotherapy may be considered, although its efficacy remains uncertain.^2^ However, the majority of patients experience recurrence, leading to poor overall survival (OS) rates.^3^ New approaches are needed to improve outcomes for OC patients. One potential strategy is early detection. Most OC are diagnosed at advanced stages, resulting in lower long‐term survival rates.^4^ Detecting OC at an early stage could lead to improved outcomes and increased survival rates. In addition to early detection, advancements in front‐line maintenance therapy have aimed to extend the interval between primary treatment and disease recurrence. For instance, maintenance therapy involving bevacizumab or PARP inhibitors has demonstrated efficacy in prolonging progression‐free survival (PFS); however, it has not yet translated into improved OS.^5^, ^6^ Despite the promising prospects of biologic immunotherapy, further research is needed to support its clinical application. Therefore, there is an unmet need for more effective maintenance therapy options for OC patients.

Histone post‐translational modifications (PTMs) play a critical role in cellular processes and the maintenance of chromatin structure. Dysregulation of histone PTMs has been extensively linked to cancer, both globally across the genome and at specific gene loci.^7^ In recent years, immunotherapy has emerged as a promising treatment for certain cancers. However, not all patients respond to immunotherapy, and identifying biomarkers to predict responsiveness is crucial for optimizing treatment strategies. Epigenetic modifications, including histone PTMs, have been implicated in cancer and immune cell dysregulation and can serve as potential biomarkers.^8^ Histones are abundant cellular proteins that can be easily assayed using high‐throughput technologies, making them attractive targets for biomarker discovery. Histone deacetylase inhibitors (HDACIs) have emerged as potential anti‐cancer drugs, with preclinical studies demonstrating promising outcomes in OC. However, clinical trials utilizing HDACIs as monotherapy have yielded mixed results and limited success.^9^ Thus, we suspected histone modification related genes might play certain role in predicting prognosis of OC patients.

In this study, we aimed to investigate the expression patterns of histone modification‐related genes in OC, construct a prognostic model, analyse their relationship with the immune microenvironment, and screen drugs. This study provides a theoretical basis for clinical practice and therapy development. We investigated the cell types present in the OC tissue microenvironment and their functional differences. We found that MIF signalling plays a crucial role in mediating fibroblast signal transduction. Additionally, we developed a histone modification‐based risk score model by identifying five key genes (CGN, LBX2, CCL18, CDC7 and ELF3) that were strongly associated with the prognosis of OC patients. This risk score was found to be correlated with immune infiltration, specifically with different types of T cells and M1 macrophages. Furthermore, we discovered that LBX2 promoted OC cell proliferation in vitro. These findings suggest that histone modification‐related genes could serve as potential biomarkers for predicting OC prognosis and may represent therapeutic targets for the development of more effective therapies.

## 2 METHODS

### 2.1 Acquisition and processing of transcriptome data

We included bulk RNA‐Seq data from 378 OC patients in the TCGA‐OV cohort (https://portal.gdc.cancer.gov/), 379 OC patients in the GSE140082 cohort (https://www.ncbi.nlm.nih.gov/geo/query/acc.cgi?acc=GSE140082), 173 OC patients in the GSE53963 cohort (https://www.ncbi.nlm.nih.gov/geo/query/acc.cgi?acc=GSE53963), and single‐cell scRNA‐Seq data from four advanced OC patients in the GSE154600 cohort (https://www.ncbi.nlm.nih.gov/geo/query/acc.cgi?acc=GSE154600) for further analysis.

### 2.2 Processing of scRNA‐Seq data and cell annotation analysis

First, we performed single‐cell analysis on four advanced OC samples from the GSE154600 scRNA‐Seq data. Based on the ‘Rtsne’ and ‘ggplot2’ packages, we employed the *t*‐distributed Stochastic Neighbour Embedding (t‐SNE) algorithm to perform two‐dimensional dimensionality reduction clustering and visualization of single‐cell sequencing data. The parameters used were as follows: ncomponents = 2, perplexity = 30, earlyexaggeration = 4.0, learningrate = 1000, niter = 1000 and niterwithout_progress = 30. Cells were categorized into six subgroups, and we also illustrated the distribution of cells from four patient samples. We used GSEA to score the enrichment of gene sets in the histone modification pathway from the GSEA database (https://www.gsea‐msigdb.org/gsea/index.jsp), dividing samples into histone modification‐high and histone modification‐low groups. We used the limma package and the eBayes function to identify DEGs between the two groups. Further analysis was then conducted on the bulk transcriptome data.

### 2.3 Functional enrichment analysis

GO analysis, KEGG analysis, and GSEA‐GO were performed using the R package ‘clusterProfiler’ (version 4.0.5), with a false discovery rate (FDR) < 0.05 considered significant enrichment. COX survival analysis was performed on DEGs using the tinyarray package, selecting genes with *p* < 0.05, which screened out a total of 299 qualified DEGs.

### 2.4 Establishment of histone modification risk score model

From the TCGA‐OV cohort, Lasso regression was used to select five prognostic genes, namely CGN, LBX2, CCL18, CDC7 and ELF3, from the 299 DEGs and construct a prognostic model. The median risk score was used to differentiate the low‐risk and high‐risk groups, and a risk score was developed. To analyse the survival difference between two risk groups, we utilized the ‘survminer’ and ‘survival’ packages to generate Kaplan–Meier survival curves for the two groups, observing the OS discrepancy. The parameter settings were as follows: surv.median.line = ‘hv’, pval = TRUE and break.x.by = 20. Independent external validation was performed using 379 OC patients from the GSE140082 cohort and 173 OC patients from the GSE53963 cohort. The value of risk score = Gene_1_* Coef_1_ + Gene_2_* Coef_2_ + … + Gene_n_* Coef_n_.

### 2.5 Immune‐related analysis and drug analysis

Immune infiltration scores were calculated using three methods: MCPcounter, ssGSEA and xCell algorithms. Visualization was performed using box plots, heat maps and scatter plots. Drug sensitivity was calculated using the ‘oncoPredict’ package in R to assess drug sensitivity in the Genomics of Drug Sensitivity in Cancer (GDSC, https://www.cancerrxgene.org/) database. We conducted a normalization check on the downloaded expression matrix, utilizing the Robust Multi‐array Average (RMA) normalization method and log transformation, resulting in values ranging from 0 to 15. Subsequently, we employed the calcPhenotype function for drug prediction, with the following parameter settings: trainingExprData = GDSC2_Expr, trainingPtype = GDSC2_Res, batchCorrect = ‘eb’, powerTransformPhenotype = TRUE, removeLowVaryingGenes = 0.2, and minNumSamples = 10. Our primary focus was to showcase the correlation between the top 37 drugs and the model genes, utilizing the Spearman correlation method. Additionally, we analysed the differences in scores for select drugs among different risk groups.

### 2.6 Cell communication analysis and tumour pathway analysis

Cell communication between classified cell types was analysed using the liana software. Various algorithms, including ‘natmi,’ ‘connectome,’ ‘logfc,’ ‘sca’ and ‘cellphonedb,’ were employed to perform this analysis. Additionally, the PROGENy model was utilized to predict the activity of tumour‐related pathways.

### 2.7 Cell culture and transfection

This study employed human normal ovarian epithelial cell line IOSE‐80, and human ovarian cancer cell lines SKOV3 and OVCAR‐3. All cells were maintained in a humidified cell culture incubator at 37°C with 5% CO_2_, and passaged every 24–36 h to sustain logarithmic growth.

For knockdown experiments targeting LBX2 in the OVCAR‐3 cell line, we employed siRNA designed and synthesized by a biotechnology company (Sangon Corporation, China). Initially, cells were dissociated from culture flasks and uniformly seeded at a concentration of 4 × 10^5^ cells per well in a 6‐well plate, with each well supplemented with complete growth medium to a final volume of 1.8 mL. Upon cell adhesion, siRNA and transfection reagent Lipofectamine 2000 (Thermo, USA) were pre‐mixed in Opti‐MEM (Thermo, USA) according to the manufacturer's instructions. After a 20 min incubation at room temperature, the mixture was evenly added to the respective wells. The medium was replaced after 4 h, and subsequent experiments were conducted 48 h post‐transfection.

### 2.8 RNA procurement and RT‐qPCR analysis

Total RNA was extracted from the samples as previously described.^10^, ^11^ The cells were dissociated from the six‐well plate using trypsin (KeyGen, China). After centrifugation for 10 min, the cell pellet was washed three times with phosphate‐buffered saline (PBS). Subsequently, 800 μL of Trizol (Takara, Japan) was added to the cell pellet for cell lysis. After a 5‐min incubation on ice, 180 μL of chloroform (SINOPHARM, China) was added to the Eppendorf tube. The mixture was vigorously shaken and then centrifuged at 4°C for 10 min. Approximately 400 μL of the supernatant was transferred to a new Eppendorf tube. An equal volume of isopropanol (Sinopharm, China) was added to the new tube, followed by a 5‐min incubation on ice and low‐temperature centrifugation. After discarding the isopropanol, an appropriate amount of anhydrous ethanol (SINOPHARM, China) was added to wash the residual isopropanol. After aspirating all liquids and drying for 20 min, an appropriate amount of diethyl pyrocarbonate (DEPC)‐treated water was added to fully dissolve the precipitate. The concentration and quality of the RNA were determined using a NanoDrop 2000 spectrophotometer. cDNA synthesis was carried out using a reverse transcription kit (MR05201M; Monad). For the subsequent PCR reaction, ChemoHS qPCR Mix (MQ00401S; Single‐celled organism) was used along with ACTIN as a reference gene and the specific primers listed below. The relative expression of transcripts was calculated using the 2^−ΔΔCt^ method. The primers used in this study are as follows (5′→3′):

GAPDH forward: ACCTGACCTGCCGTCTAGAA,

GAPDH reverse: GTCAAAGGTGGAGGAGTGGG;

LBX2 forward: CGTTTAGTGTTGCGTTAAGGGTTT,

LBX2 reverse: AAAATCGAATCTTTCCGAATAACCAAA;

ELF3, forward: CATGACCTACGAGAAGCTGAGC,

ELF3, reverse: GACTCTGGAGAACCTCTTCCTC;

CGN, forward: GACAGTTCTGCAGTCCACCA,

CGN, reverse: TAGCTGGTCCTTCTGGTCGT.

### 2.9 CCK‐8 and colone formation assay (CFA) analysis

Cell viability was assessed using the CCK8, following the manufacturer's instructions. Cells were seeded and cultured in 96‐well microplates (Corning, USA) at a density of 4 × 10^3^ cells per well in 100 μL of medium. The cells were subsequently transfected with the indicated si‐RNA, and following 48 h of treatment, 10 μL of CCK8 reagent was added to each well. This was incubated for 1 h before absorbance was analysed at 450 nm using a microplate reader (Thermo Fisher, USA), with wells without cells used as blanks. Cells were inoculated in six‐well plates at the temperature of 37°C for 9 days. Then, the cell colonies were fixed with 4% paraformaldehyde. We used the crystal violet to dye the colonies for subsequent counting.

### 2.10 Statistical analyses

All statistical analyses in this study were conducted using the R programming language. Cox regression analyses were performed using the ‘survival’ and ‘survminer’ R packages. Survival curves were generated using the ‘survminer’ package. To address the issue of multiple testing, the Benjamini‐Hochberg procedure was applied to control the FDR. We ensured the robustness of our experiments by conducting a minimum of three technical replicates for each experiment, and all results demonstrated reproducibility. A statistical significance threshold of *p* < 0.05 was adopted to determine statistical significance in our analyses.

## 3 RESULTS

### 3.1 Functional characterization of different cell types in the OC

**FIGURE 1:**
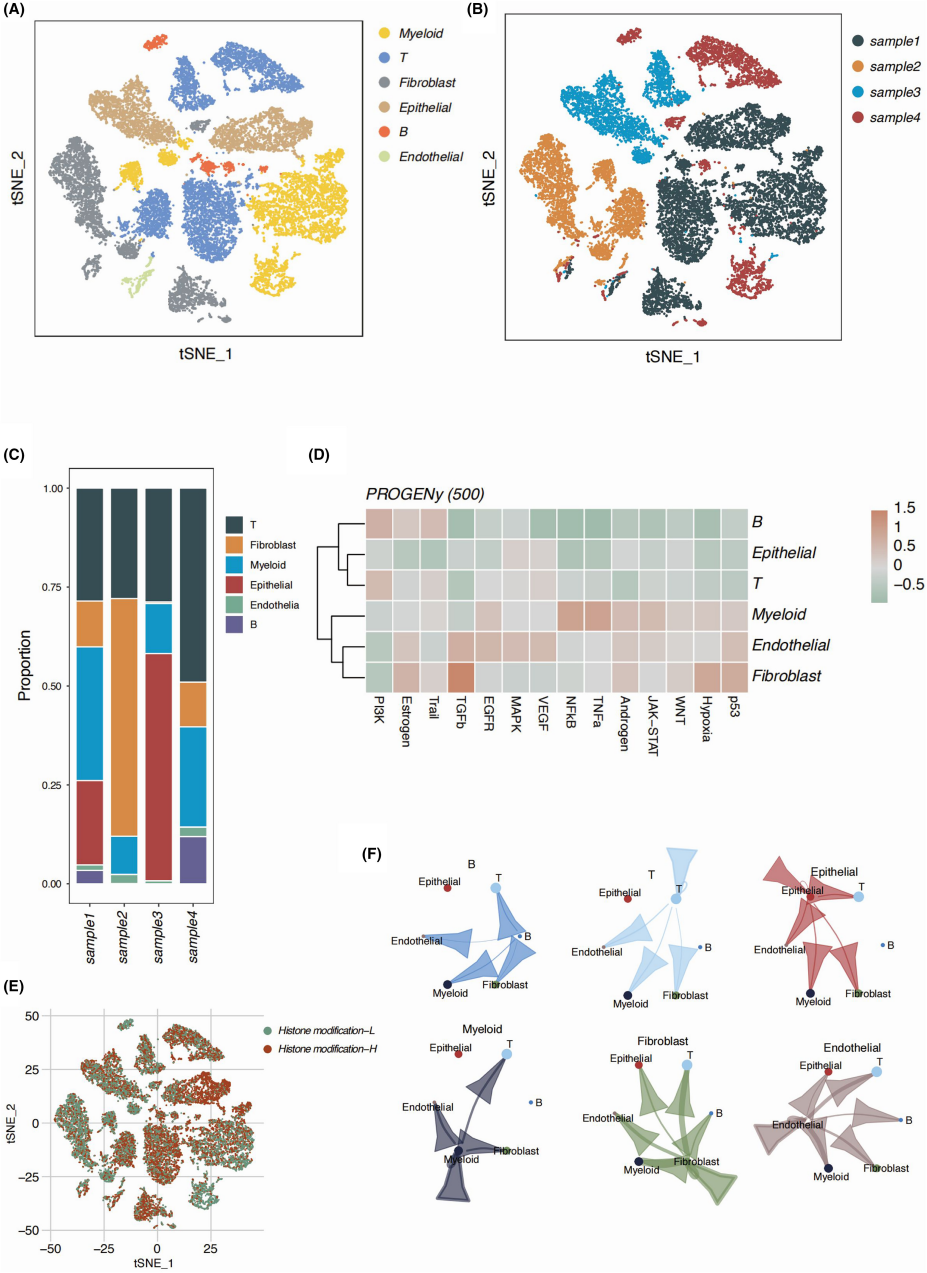
Functional characterization of different cell types in the OV cancer. (A) The t‐SNE plot of the six cell clusters obtained after dimensionality reduction and clustering. (B) The t‐SNE plot showing the cell proportions in four different patients with OC. (C) The stacked bar plot illustrating the proportions of different cell clusters in the four patients with OC. (D) The heatmap diagram showing GSVA score normalized value based on PROGENy scoring. (E) The t‐SNE plot demonstrating the cell identified in the OC patients with the histone modification gene set enrichment score. (F) The dot line plot illustrating the cellchat analysis based on the six cell subgroups.

**FIGURE 2:**
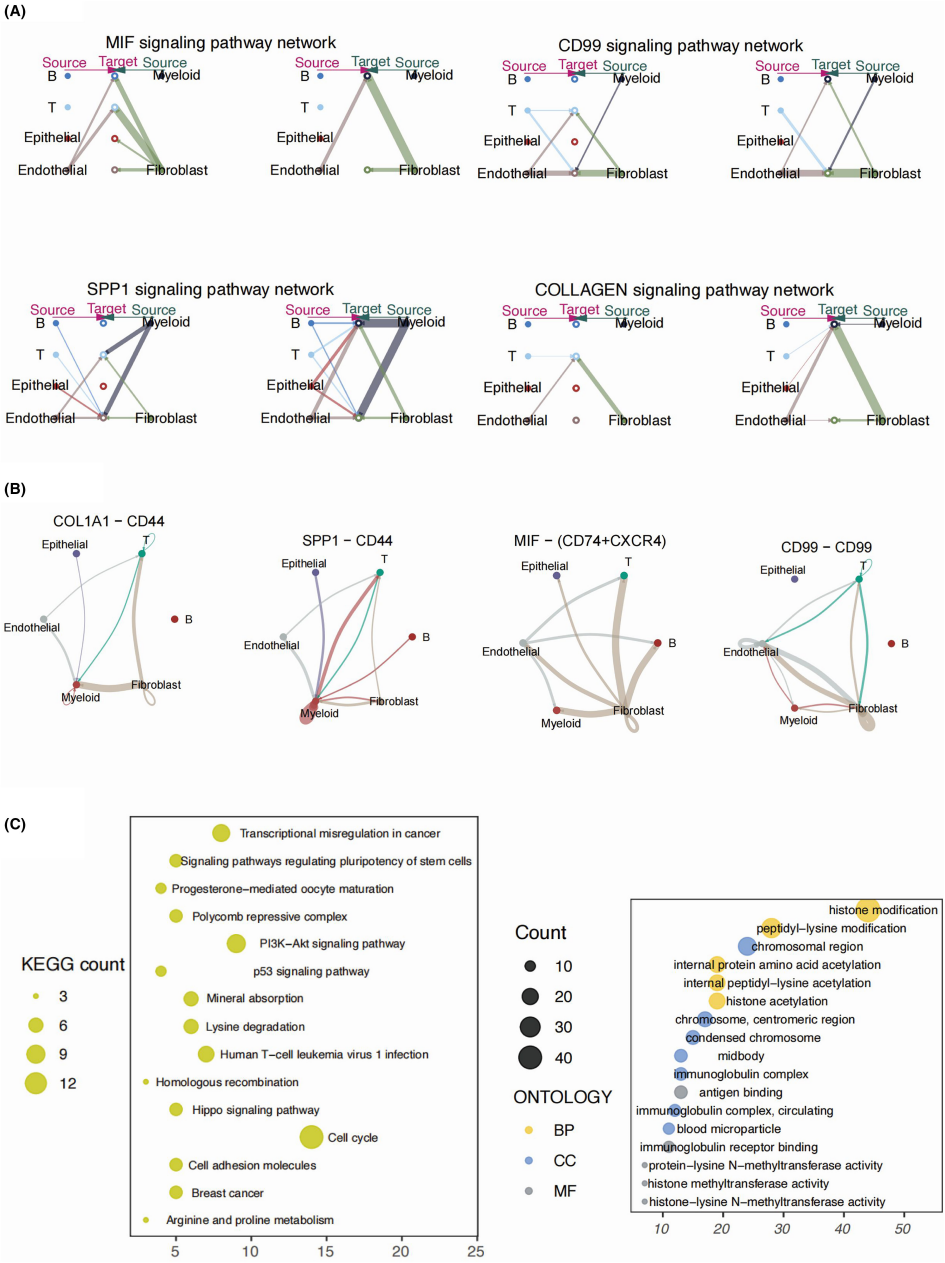
Cell communication in the OC patients and functional enrichment analysis of identified DEGs. (A) The cellchat pathway signalling plots of the CD99, collagen, MIF and SPP1 signalling pathway networks. (B) The signalling pathway analysis of CD99, collagen, MIF and SPP1 signalling pathway, shown in the form of dot line plot. (C) The dot plot displaying the GO analysis and KEGG analysis results of the identified DEGs.

Single‐cell RNA‐Seq data from four advanced OC patients in the GSE154600 cohort was enrolled in our study for detailed annotation analysis. We successfully annotated six main cell types in the advanced OC patients, namely myeloid cell, T cells, B cells, fibroblasts, epithelial cells and endothelial cells (Figure 1A,B). We found a universal composition of T cells and myeloid cells across the four OC samples. Moreover, the composition of fibroblasts proved to be highly fluid between different samples (Figure 1C). We took a deeper insight into the functional difference between different cell types in the TME of OC by resorting to the PROGENy algorithm. A distinct enrichment of TGFb activity was observed in the fibroblasts. The P53 was significantly negatively enriched in the epithelial cluster of the OC cancer, suggesting a highly proliferative phenotype possessed by the annotated OC cells (Figure 1D). We used GSEA to score the enrichment of gene sets of the histone modification pathway from the GSEA database, dividing clustered cells into histone modification‐high and histone modification‐low groups (Figure 1E). To further determine the interaction between different main cell types in OC samples, we applied the cell chat analysis. We observed a heated interaction between the myeloid cells, T cells, B cells and endothelial cells, which were consistent with role of endothelial cells as porter of infiltrating immune cells in the TME. B cells were identified as an independent cell type, sharing little to no cell‐to‐cell communication with the other cell types in the TME (Figure 1F). Fibroblasts were shown as the main regulating cell types in the OC TME by communicating with mainly endothelial cells, myeloid cells and T cells. The interaction between epithelial cells and fibroblasts were rather low, suggesting an indirect regulatory role of fibroblasts in the OC TME. MIF pathway was shown to be the main signalling pathway of the cell‐to‐cell communication originating from fibroblasts, along with COL1A1 to CD44 pathway and CD99 to CD99 pathway (Figure 2A,B). We used the limma package and the eBayes function to identify DEGs between the cells of different histone modification scores, which were further analysed by KEGG and GO analysis (Figure 2C). We found cell cycle, PI3K‐AKT pathway P53 activity and transcriptional misregulation processes were highly enriched, suggesting different extent of histone modification might be associated with regulation of these processes. In addition, most DEGs was associated with histone acetylation and condensed chromosome, which was consistent with our expectation.

### 3.2 The establishment of risk model in the OC patients

**FIGURE 3:**
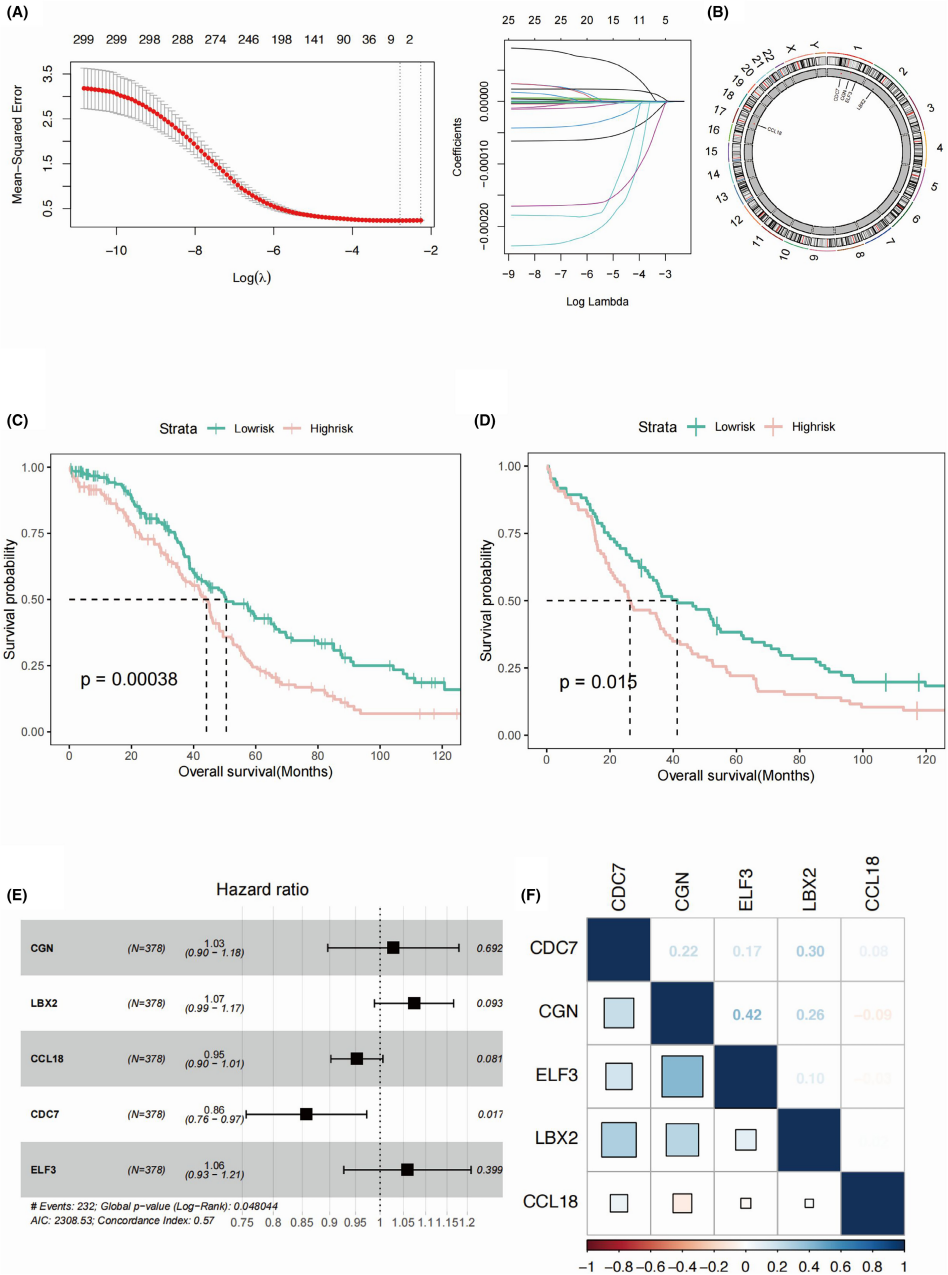
The establishment of risk model in the OC patients. (A) The Lasso regression analysis results determining the optimal key genes for prognosis predicting. (B) The distribution of the five key genes on the chromosomes. (C) The OS analysis exhibiting the survival differences of the training set of TCGA‐OV cohort, shown in the form of the Kaplan–Meier curve. (D) The OS analysis exhibiting the survival differences of the validation set of GSE53963 cohort, shown in the form of the Kaplan–Meier curve. (E) The forest plot illustrating multivariate COX analysis results of five key genes used for the establishment of prognostic model. (F) The heatmap depicting the correlation plot of the five key genes, namely CGN, LBX2, CCL18, CDC7 and ELF3.

First, we applied the LASSO regression model to determine the key genes with prognostic significance in the OC patients (Figure 3A). A total of five genes, namely CGN, LBX2, CCL18, CDC7 and ELF3, were selected from 299 DEGs to construct a prognostic model in the TCGA‐OV cohort. The value of risk score = CGN*0.0277 + LBX2*0.0710 + CCL18*‐0.0483 + CDC7*‐0.154 + ELF3*0.0572. The distribution of the five key genes was shown in the Figure 3B. We observed a distinct OS difference between OC patients of different risk score value in the TCGA‐OV as training set (Figure 3C), which generated similar outcomes in the independent external validation conducted in the GSE53963 cohort (Figure 3D). We found no single key gene could be used as independent risk factor predicting OC incidence. However, CDC7 was identified as a protective factor for OC incidence (Figure 3E). A strong positive correlation was found between ELF3, LBX2 and CGN as regard to the mRNA expression of these key genes (Figure 3F).

### 3.3 The correlation analysis targeting five key genes and immune microenvironment in OC patients

**FIGURE 4:**
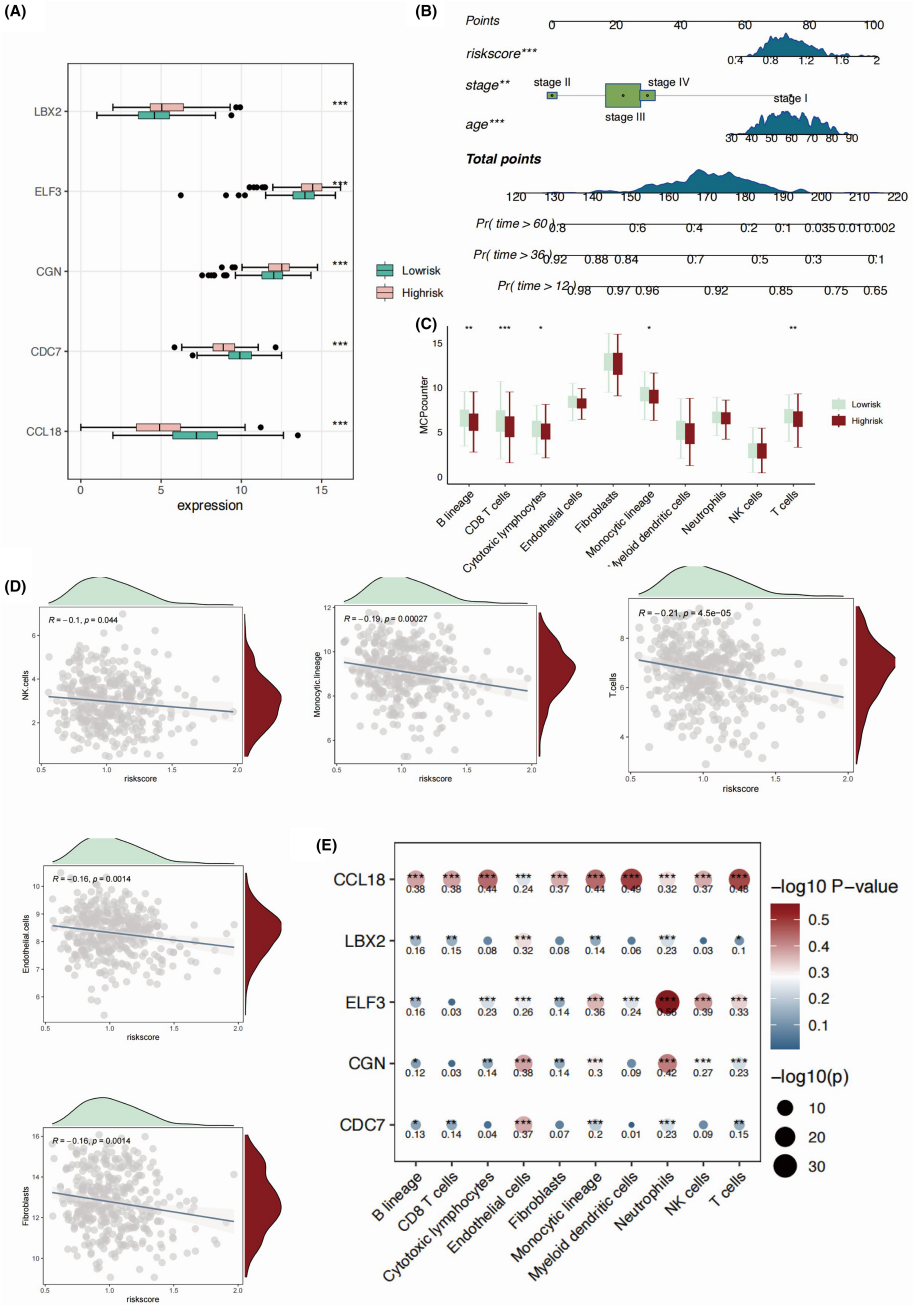
The correlation analysis targeting five key genes and immune microenvironment in OC patients. (A) The box plots showing the expression levels of five key genes in the high‐risk and low‐risk groups. (B) The nomogram integrating multiple clinical factors, including stage and age. (C) The box plots depicting the immune cell infiltration based on MCPcounter algorithm in the high‐risk and low‐risk groups. (D) Scatter plots showing the correlation between risk score and immune cell compositions in the immune microenvironment. (E) The correlation heat map showing the correlative relationship between the five key genes and immune cell infiltrations based on MCPcounter algorithm.

**FIGURE 5:**
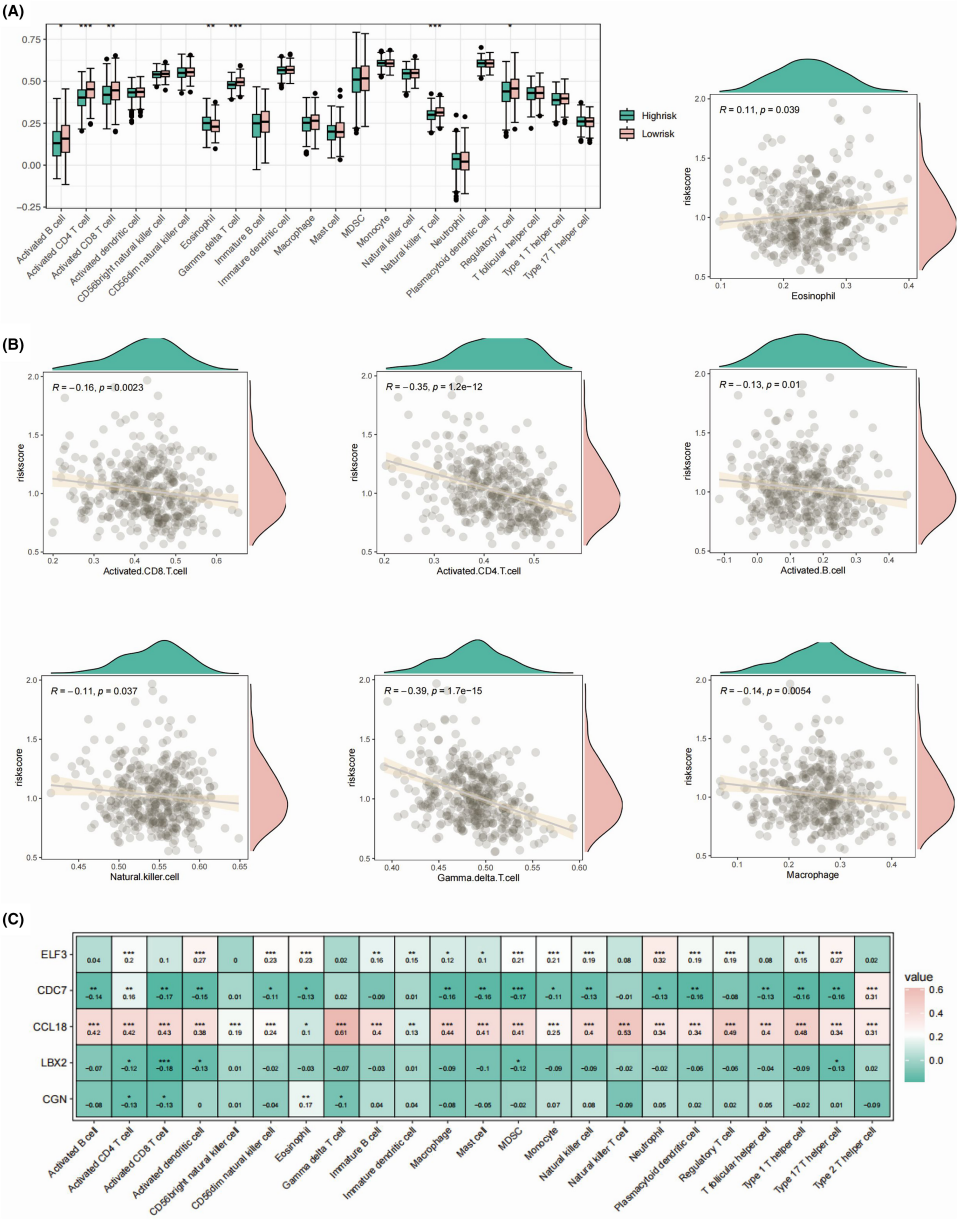
The immune and immune regulatory cells characterization in the high‐risk and low‐risk group in the OC patients based on ssGSEA. (A) The box plots showing the immune cell infiltration based on ssGSEA algorithm in the high‐risk and low‐risk groups. (B) The correlation scatter plot presenting relationships between the five key genes and immune cell infiltrations based on ssGSEA algorithm. (C) The correlation heat map illustrating the correlation between the five key genes and immune cell infiltrations based on ssGSEA algorithm.

**FIGURE 6:**
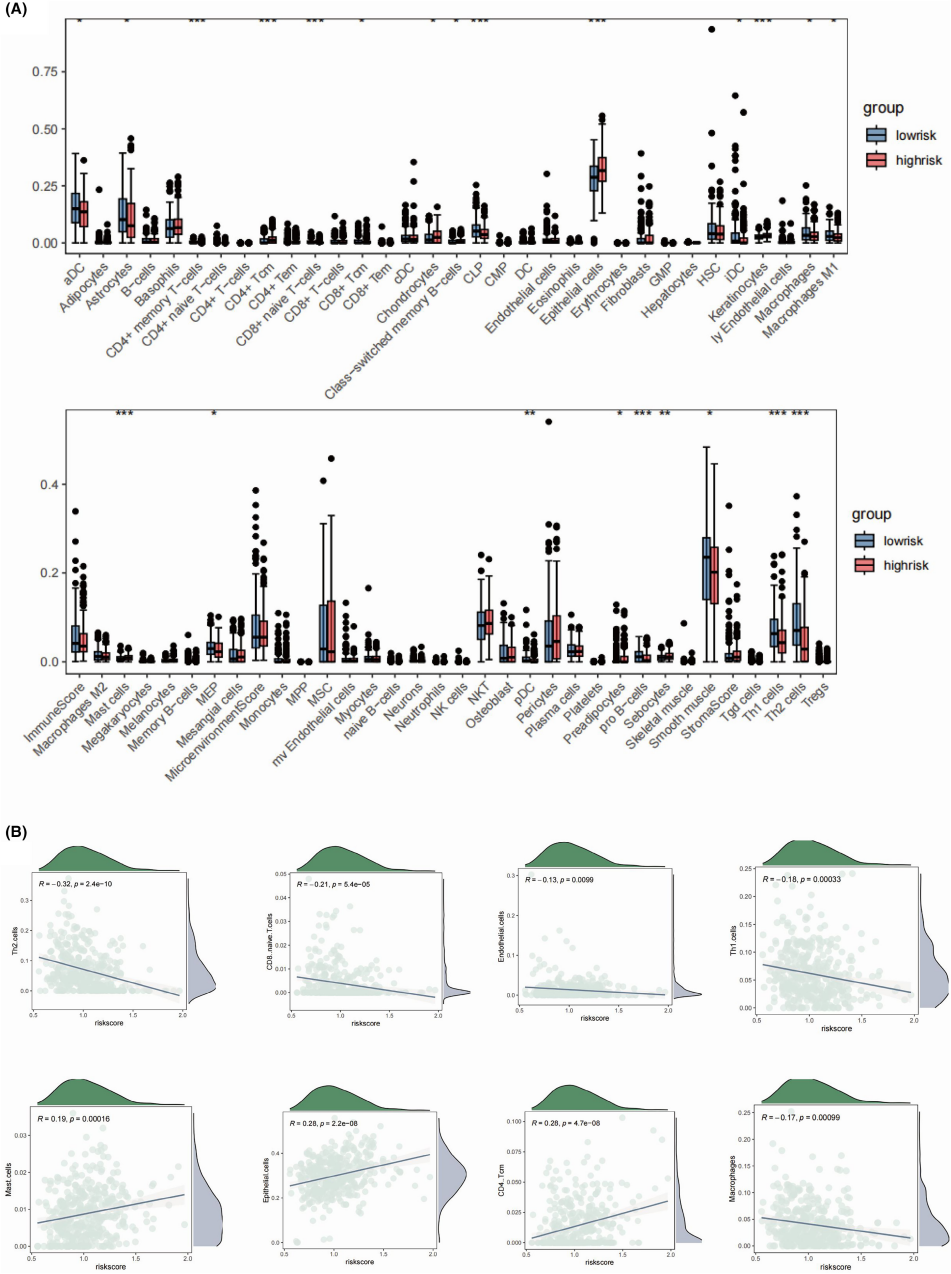
The immune and immune regulatory cells characterization in the high‐risk and low‐risk group in the OC patients depicted by xCell. (A) The box plots showing the immune cell infiltration based on xCell algorithm in the high‐risk and low‐risk groups. (B) The correlation scatter plot presenting the correlative relationship between the risk score value and immune cell infiltrations based on xCell algorithm.

**FIGURE 7:**
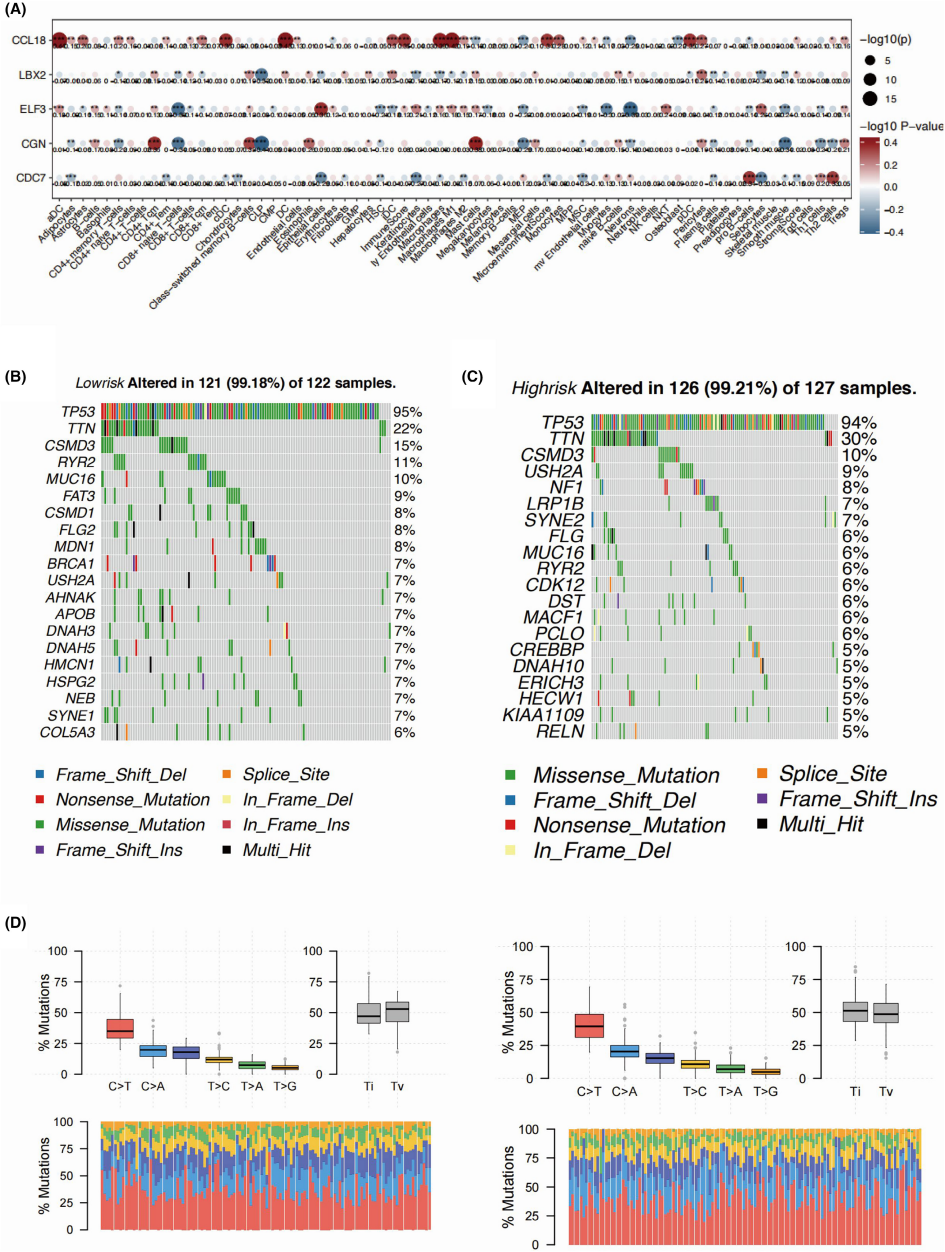
Immune and mutation landscapes in the high‐risk and low‐risk groups in the CO patients. (A) The correlation heat map illustrating the relationship between the five key genes and immune cell infiltrations based on xCell algorithm. (B, C) The waterfall plots displaying the top frequently mutated genes in the high‐risk and low‐risk groups. (D) The box plot showing the detailed mutation type in the OV cancer patients of different risk value.

We found significantly higher expression levels of ELF3, LBX2 and CGN in the high‐risk group of OC patients (Figure 4A), while CDC7 and CCL18 were both higher in the low‐risk group. We further integrated the clinical factors, including pathological stage and patient age into the consideration of prognostic model, which generated the monogram model for more accurate clinical implementation (Figure 4B). We delineated the differences in immune microenvironment in OC patients by applying MCPcounter algorithm (Figure 4C). T, B cells and monocytic cells were found to be elevated in the low‐risk group, suggesting an overall higher infiltration of immune cells in the low‐risk group of OC patients. Moreover, cytotoxic lymphocytes and CD8+ T cells were both higher in the low‐risk group, suggesting a more favourable clinical outcome. In addition, the abundance of NK cells, endothelial cells and fibroblasts were all shown to be negatively correlated with the value of risk score (Figure 4D). Among the five key genes, the expression of ELF3 was highly associated with higher neutrophil, NK and monocytic cells infiltration (Figure 4E). As a well‐established chemokine, CCL18 was identified as positively correlated with majority of immune cells in the OC TME. We applied ssGSEA to further study the detailed profile of the infiltrated immune cells (Figure 5A). Consistent with our former observation, we found higher levels of activated CD4 and CD8 T cells in the OC patients with lower risk score value (Figure 5A). Moreover, we found higher infiltration of Treg cells in the low‐risk group. Except for eosinophil, we found all the other infiltrated immune cells negatively correlated with the risk score value, especially the gamma delta T cells (Figure 5B). Among the five key genes, the CCL18 showed highest association with the infiltration of the immune cells, especially the Tregs, NKT cells, and gamma delta T cells (Figure 5C). Macrophage and M1 macrophage were shown to be elevated in the low‐risk group, while M2 macrophage showed no significant differences, indicating a pro‐inflammatory role of M1 macrophage infiltrated mediating the clearance of the M1 macrophage (Figure 6A). Epithelial cells were found higher in the high‐risk group, while different types of DCs were all elevated in the low‐risk group of OC patients. Epithelial cells were shown to be significantly correlated with risk score value (Figure 6B). In addition to CCL18, ELF3 was identified as a key gene, which might regulate the infiltration of the macrophages (Figure 7A).

### 3.4 Detailed mutation and drug sensitivity profile in the OC patients of different risk score value

**FIGURE 8:**
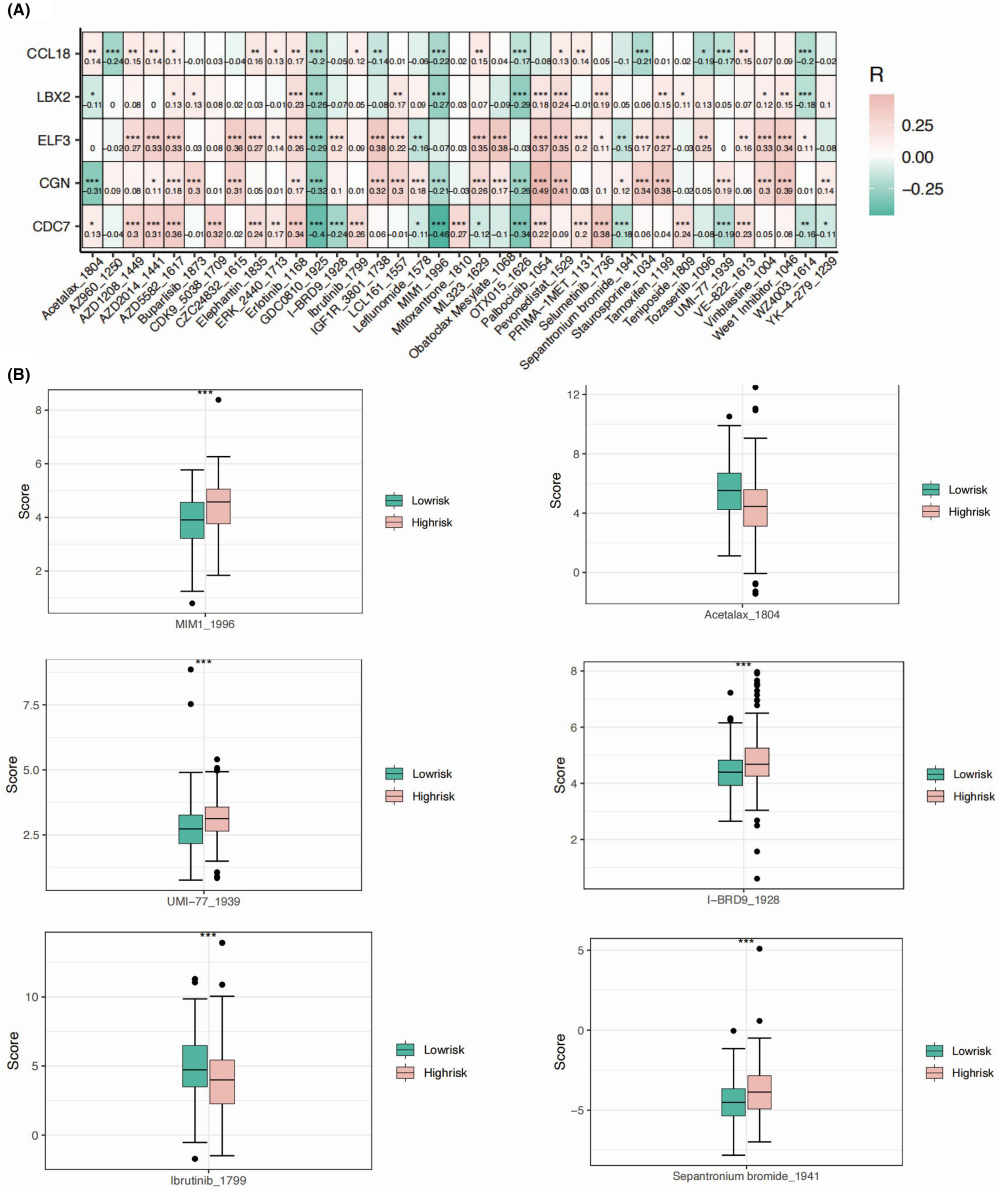
Drug sensitivity analysis showing the optimal drugs for OC patients of different risk score value. (A) The correlation heat map presenting the relationship between the five key genes and sensitivity‐related drugs. (B) The box plots displaying the drug sensitivity scores in the high‐risk and low‐risk groups.

We found the same top three most frequently mutated genes in the two risk groups, namely TP53, TTN and CSMD3, with slightly different mutation rate (Figure 7B). The FAT3 showed a markedly higher rate of mutation in the low‐risk group, while no significant mutation incidence was found in the high‐risk group (Figure 7C). The detailed mutation types were found highly similar between two risk groups (Figure 7D). We calculated the contribution of different key genes in the predicted response to different drugs. CDC7 showed the significant negative correlation with MIMI_1996, GDC0810_1925, and OTX015_1626 (Figure 8A). CGN showed highest positive correlation with sensitivity to Palbociclib_1054 and Pevonedistat_1529. Both the Ibrutinib_1799 and Acetalax_1804 were found with more favourable response in the low‐risk group of OC patients, while MIMI_1996 and UMI_77_1939 might be more sensitive to OC patients with higher risk value (Figure 8B).

### 3.5 LBX2 promoted OC cell proliferation in vitro

**FIGURE 9:**
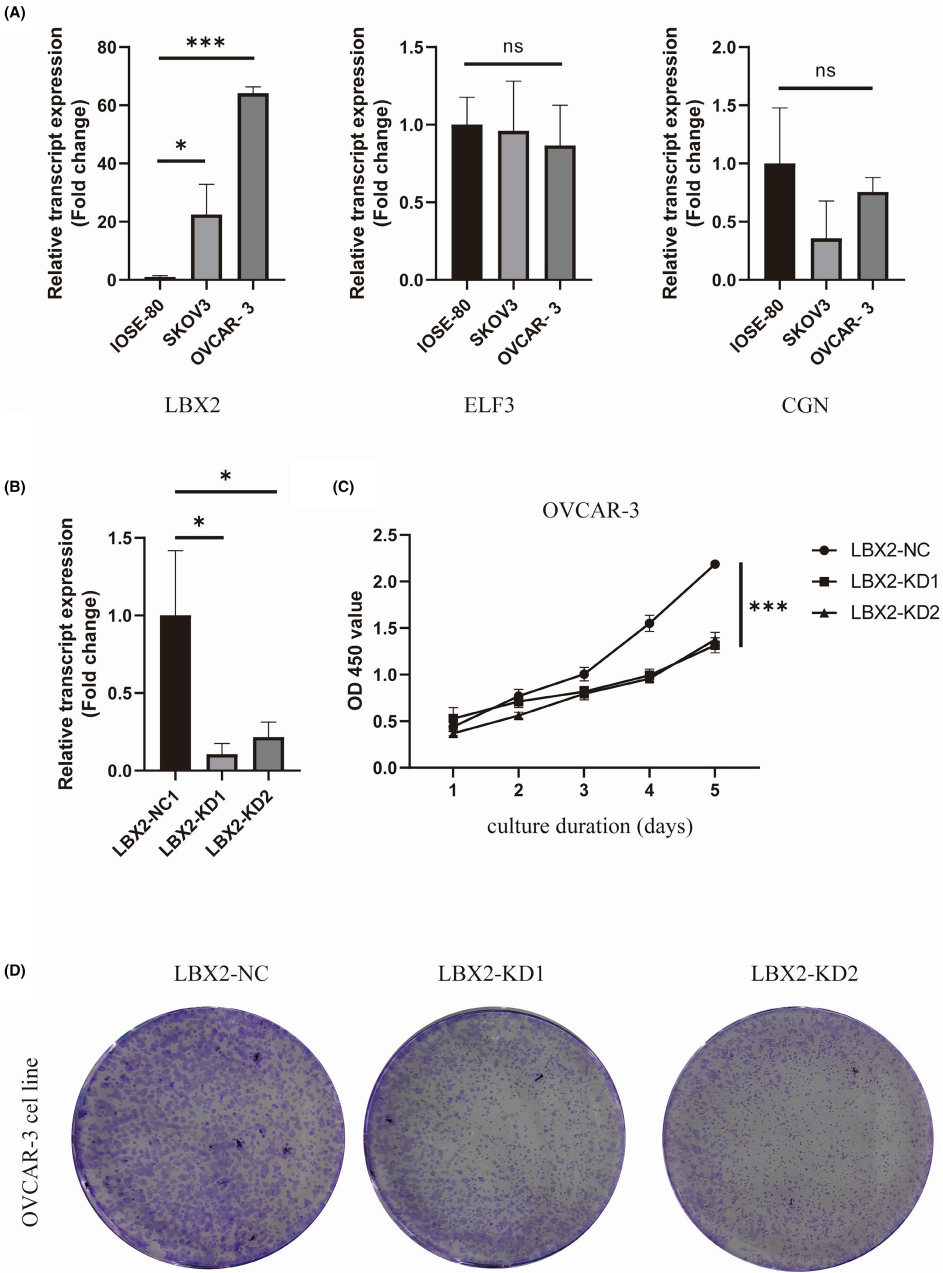
Identification of LBX2 as a proliferation promoter gene in vitro. (A) Screening the mRNA expression levels of all the possible risk factor genes in the normal ovarian epithelial cells and cancerous cell lines. (B) The verification of LBX2 knockdown by assessing the mRNA level in the OVCAR‐3 cell line. (C) The knockdown of LBX2 significantly reduced the proliferation rate of OVCAR‐3 and LBX2‐knockdown cell lines. (D) CFA analysis results of normal and LBX2‐knockdown OVCAR‐3 cell lines. *P<0.05; ***P<0.001.

We observed a distinguishable up‐regulation in the expression level of the LBX2 in high‐risk group. We assessed all three key genes using RT‐qPCR in the normal ovarian epithelial cells and cancerous ovarian epithelial cells in vitro. The mRNA expression level of LBX2 in the IOSE‐80 cell line was significantly lower than the cancerous cell lines of SKOV3 and OVCAR‐3 cell lines (Figure 9A), while ELF3 and CGN showed no significant differences. The knockdown of LBX2 was verified by the significantly reduced mRNA level (Figure 9B). We observed a distinguishable reduction in the proliferation of OVCAR‐3 cell line after LBX2 knockdown, especially at the time point of Day 5 (Figure 9C). The reduced proliferation rate of OVCAR‐3 cell line was further verified by consistent CFA results, indicating knockdown of LBX2 largely inhibited the growth of OVCAR‐3 cell line. Collectively, we identified LBX2 as a potent promoter of OC cell proliferation in vitro.

## 4 DISCUSSION

We identified different cell types in the OC tissue microenvironment and characterized their functional differences. We established a histone modification‐based risk score model by screening out five key genes (CGN, LBX2, CCL18, CDC7 and ELF3) that were associated with prognosis of OC patients. We found that the risk score was correlated with immune infiltration of different types of T cells and M1 macrophages. Histone modification‐related genes may serve as potential biomarkers for predicting the prognosis of OC patients. They also highlight the potential of these genes as therapeutic targets for the development of more effective therapies. Moreover, our study identifies LBX2 as a novel cell proliferation promoter in the carcinogenesis of OC. These findings contribute to our understanding of the molecular mechanisms underlying OC and provide insights for the development of targeted therapies.

The ELF3 transcription factor, a member of the epithelium‐specific ETS (ESE) family, plays a crucial role in regulating gene expression and maintaining the integrity of epithelial tissues.^12^ Mutations in ELF3 have been identified in various types of cancers, including bladder, cervical, ovarian and gastrointestinal cancers. Inactivating mutations of ELF3 have been particularly observed in approximately 6% of mucinous OC, indicating their potential involvement in OC development.^13^ Loss of ELF3 mRNA and protein expression has been associated with a worse prognosis in OC, suggesting that ELF3 may act as a tumour suppressor in this context.^14^ Experimental studies have demonstrated that re‐introducing ELF3 expression in OC cell lines with low endogenous expression inhibits cell proliferation both in vitro and in vivo, supporting its tumour‐suppressive role. Additionally, the restoration of ELF3 expression promotes a transition from a mesenchymal to an epithelial state, a process known as mesenchymal to epithelial transition (MET). On the other hand, knocking down ELF3 expression in OC cell lines induces epithelial to mesenchymal transition (EMT), a phenomenon associated with enhanced tumour progression and metastasis.^14^ These findings indicate that ELF3 plays a critical role in maintaining the epithelial state and inhibiting the progression of ovarian tumours. Further investigation into the underlying molecular mechanisms by which ELF3 exerts its tumour‐suppressive effects may provide valuable insights into the development of targeted therapies for OC and other cancers with aberrant ELF3 expression.

CCL18, a chemokine belonging to the β‐chemokine sub‐family, is characterized by the presence of a ‐Cys‐Cys‐ motif at the N‐terminus.^15^ It shares a sequence homology of 59% with a protein and approximately 50% with the cDNA for CCL3/macrophage inflammatory protein 1α (MIP‐1α), suggesting that the CCL18 gene may have arisen from the duplication and fusion of two MIP‐1α‐like genes.^16^ Studies conducted in vitro and in vivo have shown that CCL18 is produced in significant amounts by tumour‐associated macrophages (TAMs)^17^ in the tumour microenvironment. It is also produced in smaller amounts by cancer‐associated fibroblasts (CAF)^18^ and cancer cells,^19^ such as colon cancer cells. CCL18 has been found to play a role in tumour cell proliferation, although its effects seem to vary depending on the type of tumour. For instance, in non‐small cell lung cancer cells^20^ and pre‐B acute lymphocytic leukaemia cells,^21^ CCL18 has been shown to reduce cell proliferation. Conversely, in OC, CCL18 has been found to increase cancer cell proliferation.^22^ One of the receptors for CCL18 is PITPNM3, which has been extensively studied in the context of CCL18‐dependent migration induction, invasion, and EMT in tumour cells. PITPNM3 is known to play a crucial role in the migration and metastasis of OC cells.^23^ These findings shed light on the complex role of CCL18 in cancer and highlight the importance of considering the specific tumour context when studying its effects on cell proliferation and migration. However, in our study, we failed to identify CCL18 as an independent risk factor for the OC incidence, which could be largely attributed to the limited size of cohort. Further research is needed to fully understand the molecular mechanisms underlying CCL18 signalling and its potential as a therapeutic target in OC.

The LBX2 protein belongs to the homeodomain‐containing family of transcription factors, known for their significant involvement in various biological processes. In zebrafish, LBX2 has been implicated in the regulation of hypaxial myogenic precursor cell migration and muscle cell differentiation.^24^ Additionally, LBX2 deficiency has been shown to disrupt the normal development of the heart by affecting the migration of neural crest cells and the process of cardiac septation.^25^ However, despite these findings, limited information exists regarding the oncogenic role of LBX2. The association between LBX2 expression and colorectal cancer (CRC) carcinogenesis was depicted in the study published by Huang et al. Overexpression of LBX2 was found to be associated with the development of CRC and might serve as a novel prognostic marker and therapeutic target in CRC.^26^ LBX2 contributed to lung adenocarcinoma (LUAD) cell proliferation, migration, and invasion through the induction of EMT progression, indicating an oncogene role in the LUAD in vitro.^27^ We suspected that LBX2 played a critical role in OC progression, given that LBX2 was found to be highly elevated in the high‐risk group of OC patients. Through knockdown of LBX2, we found a distinguishable reduction in the cell proliferation capability by both CCK8 and CFA analysis. These evidences were consistent with the role of LBX2 in the LUAD. The exact mechanism underlying the LBX2 mediated OC cell proliferation remained to be determined. However, our research on LBX2 provides a novel theoretical basis for the development of subsequent targeted drugs.

Our study identified a crucial role of macrophage migration inhibitory factor (MIF) in CAF signalling. We found that MIF mediated signalling originating from CAFs. The downstream pathways activated by MIF include the mitogen‐activated protein kinase (MAPK), phosphoinositide 3‐kinase (PI3K), and nuclear factor kappa B (NF‐κB) pathways, which are frequently involved in cancer progression and are subject to epigenetic regulation.^28^, ^29^ MIF has been implicated in various aspects of cancer, including cell proliferation, tumorigenesis, and metastasis. Overexpression of MIF has been observed in different types of tumours, such as genitourinary cancer,^30^ melanomas,^30^ and head and neck cancers.^31^ A recent meta‐analysis revealed that MIF overexpression is associated with a poor prognosis and lower survival rates in cancer patients. Mechanistically, MIF activates the MAPK and PI3K pathways, which are crucial for regulating essential cellular functions, including cell proliferation, differentiation, apoptosis, and survival.^32^ These pathways are also known to play a role in cancer development and progression. Our findings highlight the importance of MIF in CAF signalling and its potential as a prognostic factor and therapeutic target in cancer. Further research is needed to fully elucidate the molecular mechanisms underlying MIF‐mediated signalling and its implications in cancer biology.

TP53, TTN and CSMD3 are consistently among the top three genes with the highest mutation frequencies in both risk groups. However, there are slight differences in mutation rates between the two groups. This consistency suggests that these genes may play pivotal roles in the tumorigenesis of both low and high‐risk groups. FAT3 exhibits a significantly higher mutation rate in the low‐risk group compared to the high‐risk group, while no significant mutation occurrence is observed in the high‐risk group. This disparity implies that FAT3 mutations may have a more pronounced impact on the tumorigenesis or progression of ovarian cancer in the low‐risk subgroup. While certain genes may exhibit differential mutation frequencies, the overall mutation spectrum remains consistent across both risk groups. The observed mutation patterns may reflect underlying differences in the molecular pathways or biological processes driving ovarian cancer development and progression in distinct risk groups. Understanding the distinct mutation profiles and their association with different risk groups may have implications for risk stratification, prognosis, and personalized treatment approaches in ovarian cancer patients. Further investigations into the functional significance of identified mutations and their impact on tumour biology are warranted to elucidate underlying mechanisms and guide the development of targeted therapeutic strategies.

The implications of drug sensitivity analysis are significant for potential therapeutic interventions in OC. First, identifying drugs that effectively target the model genes associated with histone modification could lead to the development of more precise and personalized treatment strategies. Second, understanding the sensitivity of OC cells to these drugs provides insights into potential therapeutic options for different patient subgroups, particularly high‐risk individuals who may require more aggressive treatment approaches.

Our study provides a novel perspective on the prognostic assessment of OC patients, revealing the potential role of key genes associated with histone modification. These genes are implicated in immune cell infiltration and signalling transduction within the tumour microenvironment, closely correlating with the survival rates of OC patients. First, we established a risk score model for histone modification‐related genes that effectively predicts the survival of OC patients. Five selected key genes (CGN, LBX2, CCL18, CDC7 and ELF3) demonstrated significant prognostic value in OC patients. Second, we observed a close association between the expression levels of these genes and the infiltration status of various immune cells, emphasizing the critical role of the immune system in OC and suggesting potential avenues for exploring immunotherapy in the future. Additionally, our research unveiled the proliferative role of LBX2 in OC cells, presenting a novel potential target for future therapeutic interventions. In summary, our study provides a fresh perspective and hope for the prognostic assessment and treatment of OC, offering important insights for the development of personalized treatment strategies.

Despite the valuable insights gained from our study, several limitations should be acknowledged. First, the retrospective nature of our analysis using publicly available datasets may introduce bias and limit the generalizability of our findings. Furthermore, while we identified potential biomarkers and therapeutic targets, further functional studies and clinical validations are necessary to confirm their roles in ovarian cancer. Moreover, our in vitro experiments were limited to a few cell lines, and the complexity of the tumour microenvironment was not fully recapitulated. Future studies incorporating larger cohorts, functional assays in relevant animal models, and clinical validation are needed to address these limitations and translate our findings into clinical practice.

## 5 CONCLUSION

In conclusion, our study sheds light on the potential prognostic significance of histone modification‐related genes in OC. Through comprehensive transcriptome analysis and functional validation, we identified five key genes (CGN, LBX2, CCL18, CDC7, and ELF3) associated with OC prognosis, providing insights into novel biomarkers for patient stratification. We elucidated the oncogenic role of LBX2 in promoting OC cell proliferation in vitro, suggesting its potential as a therapeutic target. Furthermore, our findings highlight the role of MIF signalling in mediating fibroblast signal transduction within the OC microenvironment. Our study contributes to a better understanding of OC pathogenesis and offers potential avenues for prognostic prediction and therapeutic intervention.

## AUTHOR CONTRIBUTIONS

**Jian Xiong:** Conceptualization (equal); funding acquisition (equal); writing – review and editing (equal). **Hongyuan Liang:** Formal analysis (equal); methodology (equal); writing – original draft (equal). **Xiang Sun:** Funding acquisition (equal); project administration (equal); writing – original draft (equal). **Kefei Gao:** Conceptualization (equal); funding acquisition (equal); project administration (equal); writing – review and editing (equal).

## FUNDING INFORMATION

This study was funded by the Research foundation of Guangzhou Women and Children's Medical Center for Clinical Doctor (2021BS044, 2020RC003), Plan on enhancing scientific research in GMU (02‐410‐2302169XM), and the Science and Technology Program of Guangzhou, China (2023A04J1244, 2024A03J0807, 2023A03J0881, 2024A03J0956).

## CONFLICT OF INTEREST STATEMENT

The authors note that there were no financial or commercial relationships that would have raised questions about a conflict of interest during the research's execution.

## Data Availability

The data that support the findings of this study are available from the corresponding author upon reasonable request.
